# Rhazes’ views on qualifications of physicians, a historical review

**DOI:** 10.34172/aim.2022.77

**Published:** 2022-07-01

**Authors:** Narges Tajik, Mohammad Hashemimehr

**Affiliations:** ^1^Department of Medical History, Tehran University of Medical Sciences, Tehran, Iran; ^2^Department of Medical History, Babol University of Medical Sciences, Babol, Iran

**Keywords:** Al-Hawi Fi Al-Tibb, Medical history, Physicians Exam, Rhazes

## Abstract

Testing physicians and determining their professional qualifications have been significant issues in the educational and medical system of the Islamic civilization. The purpose of this study is to explain the views of *Rhazes* on how to test physicians in the book *Al-Hawi Fi Al-Tibb*. This library study has been done with descriptive-analytical method and using the keywords of medical test, medical ethics and medical history. *Rhazes* emphasizes various criteria by holding a comprehensive test to determine the competence of physicians with the aim of evaluating different aspects of their knowledge and attitude. He enumerates the provisions of the test in three sections: individual characteristics, theoretical and practical medical sections. The results show that *Rhazes* paid attention to all aspects related to a doctor’s personal and social habits and behavior and his relationship with the patients. A number of post-*Rhazes* physicians have also mentioned to the test of physician before hiring them but their content does not have the coherence of *Rhazes’* statements. Most of the material mentioned in *Al-Hawi Fi al-Tibb* is still worth rethinking after hundreds of years. It is suggested that medical students and physicians use the ethical and professional points mentioned by this great scientist in his valuable book in order to make the high position of medical science more visible.

## Introduction

 Determining the competence of physicians and issuing licenses for medicine has been one of the most important and significant issues in the educational and medical systems in different periods of medical history.^1^ In ancient Iran, the duties of a seeker of medical science were very clearly defined.^2^

*Avesta* historical documents have indicated the advanced state of medicine, and medical services of physicians were offered in the form of various specialties such as surgery, ophthalmology, psychiatry, forensic medicine and herbal medicine. In fact, the skill of treating was one of the techniques and professions on which there was a lot of sensitivity so that its holders should have the necessary conditions and have the right to be called a physician and to be among the physicians.^3^

 According to *Zoroastrian* principles, a physician, in addition to acquiring knowledge in the presence of medical professionals, must be a reader, have a good memory, be interested in the medical profession, experienced and kind. Such a person must consciously treat and listen to his patient’s pain with patience and not work for money and material gain, be God-fearing and have information about medicines.^4^

 An acceptable physician in the Iranian society is a correct user of prayers, a prolific experimenter, an accurate man, a physiologist, an expert in the treatment of diseases, a patient assistant, free from envy, owner of a pleasant voice, free from degrading behavior, patient, nurse of children and women, skillful and a therapist.^5^

 It is stated in the medicine of the ancient Iranians: The physician must have passed the necessary tests of his skill and dexterity and he must have succeeded in treating three *non-Ahura Mazdians* in order to be able to treat one of *Ahura Mazda’s* followers. If all three died during treatment, they would not be allowed to practice medicine for life. The doctor had to visit his patient every day when necessary.^6^ In fact, he who practices medicine “not tested and without permission”, if the patient under his care is well, he will not be paid and if he injures the patient during the treatment, his punishment is “wound compensation”^5^ and he should pay blood money and retribution.^7,8^

 During the Sassanid era, physicians were given a license which is similar to today’s testimonies or certificates so that people who are entitled to medicine can practice treatment.^9^

 In relation to the signs of obtaining medical permission and passing from the student stage to the practical and clinical field in other civilizations, we can mention the Hippocratic Oath. Over the ages, the Oath of Hippocrates has been accepted as one of the major sources for medical ethics, as a “taken-for-granted ethical system”^10^ and the most enduring document of Western medicine. Hippocrates was the first who referred to ethical principles and asserted that the purpose of medicine is to protect the interests of the patient.^11^ The Hippocratic Oath was a short but elegantly complete document that for the first time defined a moral code for medical behavior^12^ and distinguished professional expertise from personal morality in medicine.^13^

 It establishes the general moral conduct of the physician–patient relationship which reflects long-lasting ethical values that still govern the medical profession. This Oath introduces the principles of beneficence, non-maleficence and confidentiality and asks for accountability from the medical community.^14^

 Besides the Hippocratic Oath, other ancient cultures also formulated oaths. One of the oldest is the oath of initiation of medical students found in the *Charaka Samhita*, (a medical manuscript of ancient India circa 4th century BC).^15^ Later on, in the 6th century AD, the Oath of *Asaph* appeared in the Middle East; it was part of the oldest Hebrew medical text, written by the Jewish physician Asaph, a disciple of the Hippocratic School that reveals many similarities to the Hippocratic Oath.^16^

 Decades later, in the 7th century AD in China, the first ethical codes appeared in the monograph of great physicians written by Sun Szu-Miao. The regulations emphasized the need for thorough medical education, rigorous conscientiousness and self-discipline.^17^ These rules were completed throughout history and we saw the first modern code of medical ethics in the Western world published in 1803 by the British physician Thomas Percival (1740-1804). He was the first to coin the term “medical ethics”.^18^

 It was as late as 1804, in the aftermath of the French Revolution, that medical graduates of the University of Montpellier recited the Oath in Latin.^19^ In the 18th century, the Oath was translated into English and medical schools in both Europe and the United States began to use various translated versions of the Oath in their graduation ceremonies.^20^

## Rules of recruiting Doctors in the Islamic Period

 In the history of medicine in Islamic civilization, determining the professional qualifications of physicians was considered one of the important issues in the educational, medical and governmental system. Physicians under the supervision of the rulers were evaluated by special groups for the necessary knowledge and skills and were allowed to practice medicine only if they were qualified.^21,22^

 It is not possible to judge for sure whether the medical students had to take any exam before they started practicing medicine but we know that there was no such exam in Baghdad before 931 AD, when the caliph *Al- Muqtadar Bellah*, The 18th Abbasid Caliph, (929-932 AD) realized that the ignorance of one of the doctors had caused the death of the diseased and for this reason, he ordered the senior city Judge to announce that whoever wants to practice medicine must first pass the exams conducted by *Sanan ibn Sabit ibn Qora*, scientist of the tenth century (880-943 AD). The Caliph personally wrote the decree and Sinan relying on this command tested the candidates and allowed them to work if he knew they had enough information. It is said that the number of people who were able to pass the relevant exam within a year after the issuance of this decree was 860. This law was implemented until three hundred years after its enactment and in recent years, *Amin al- Dawlah ibn Tilmi*z, scientist of 11th and 12th centuries (1074-1165 AD) was the chairman of the Board of Examiners.^4^

 We do not know for sure whether there was a board of examiners until the end of the caliphate. *Dr. Ahmed Issa Beyk* (1876-1946) mentions two certificates issued in Cairo in the 17th century in the book *History of Hospitals*, one for an ophthalmologist and the other for a surgeon.

 In Mesopotamia, in addition to the Board of Examiners, there was another rule to ensure adequate education for school and university students and it was that all educational institutions were under the direct supervision of the Caliph and in most cases he appointed one of the caliphate court physicians or one of the city’s top scholars as the high director of these schools and selected a person called an officer to inspect the affairs.

 According to available documents during the *Safavid* era in Iran, such a custom was also common and one of the duties of the officer in Iran was to supervise the execution of the *Hippocratic Oath* by physicians according to which physicians swore not to make poison for others or avoid giving the poison to non-specialists. Doctors swore not to give abortion drugs to pregnant women and sterilizing drugs to men. They swore not to look down on patients with temptation when visiting patients and never to reveal their patients’ secrets to anyone.^4^

## Some Examples of Exams of Different Specialists in the Islamic Period

 According to historical reports left over from the Islamic civilization, all disciplines related to medicine had to pass a test related to their field like surgeons who underwent other special tests, as well. In this way, they were asked before anything else to know the seven book of Galen’s Anatomy and they were also tested for anatomy. Surgeons also had to be equipped with the necessary surgical equipment.

 Orthopedists were another discipline that was evaluated in the Islamic civilization and they were tested on the book by *Paul of Aegina* (625-690 AD) *“the father of early medical books”* which was translated into Arabic and was about bones.

 Another discipline pertained to ophthalmologists who like surgeons underwent a special examination; before anything else, they first had to read the book *Al- Ashar al- Maqalat fi al-Ain* (Ten articles about the eye) by *Honain ibn Isaaq* (809-873 AD) and as long as they were unaware of the general dissection of the eye, they were not allowed to perform eye surgery. One of the special examinations that was administered to ophthalmologists was that they had to know enough about some of the main eye diseases and their side effects and be able to make ophthalmic drugs especially *Sorme* (black powder obtained from ferrous sulfur or lead sulfur and used to darken eyelashes and eyelids). They had to swear not to give their surgical instruments to those who were not ophthalmologists.^4^

 Studying and dealing with physicians’ examinations and screening in order to ensure the individual’s competencies and his evaluation for the treatment of patients is one of the necessities of conducting the present study. In this study, an attempt has been made to use the views and theories of *Abu Bakr Mohammad ibn Zakaria al-Rhazes*, a famous physician and scientist of the ninth and early tenth centuries, using his valuable work *Al- Hawi fi al- Tibb* to examine the conditions and criteria of physicians and their admission methods.

## A Brief Biography of Mohammad ibn Zakaria al-Rhazes

 Abu Bakr Mohammad ibn Zakaria Razi (Latin: *Rhazes* (865-925 AD)^23^ was an Iranian physician, alchemist, philosopher, astronomer and a full-fledged scientist.^24-26^ *Rhazes* who was a comprehensive thinker, had a fundamental and lasting contribution to various scientific fields and has written more than 200 scientific works.^27^ An early proponent of experimental medicine, he served as chief physician at Baghdad and Ray hospitals. His medical works and ideas became popular among medieval European physicians and had a profound effect on medical education in the West.^28^ Edward Browne describes him as “the greatest and noblest Muslim physician and one of the most prolific writers”.^29^ The most important medical work by *Rhazes* is the book *Al- Hawi Fi Al- Tibb*.

 The present research is a library study using the descriptive-analytical method. This study was performed based on the collected information using the keywords of medical exam, physician, medicine, medical ethics and history of medical sciences. For this purpose, the chapters related to the title and subject of discussion in the book *Al- Hawi Fi Al- Tibb* by *Abu Bakr Mohammad Ibn Zakaria Razi* were studied and notes were taken. Also, valid databases such as Scopus, PubMed, Science Direct and Google Scholar were searched for more information and finally, the results were presented in writing after classification and analysis.

## Physician and Medicine Exam from Rhazes’ Point of View

 There are a number of methods of letter for testing the qualifications of physicians in the works of medical scholars in Islamic civilization such as *Mohammad ibn Zakaria al-Razi*. In his book *Al- Hawi Fi Al- Tibb*, *Rhazes*, the great Iranian scientist and physician describes a comprehensive and specialized test to determine the competence of physicians which looks at various aspects of physicians’ knowledge, attitudes, ethics and behavior. This book has evaluated the physician’s experience and clinical skills and emphasized the physician’s ability to differentiate between diseases.^1^

 He was well aware of the difficulties that exist in diagnosing some diseases with similar complications, so we see that he emphasizes the need to test the physician to diagnose diseases in comparison with each other.^30^ Regarding other fields such as pharmacy, he has also mentioned a special test.^31^

 From *Rhaze’s* point of view, the conditions and criteria for testing a medical seeker and a physician can be evaluated in three parts: the individual characteristics of the physician (physical, moral and behavioral health), the theoretical part of medicine and the practical part.

## Personal Characteristics of the Physician

*Rhazes* did not ignore the smallest issues related to doctors that could have been effective in treating and benefiting the patient and pointed out some hints from taking care of appearance to communicating with patients including the notions that the doctor should be agile, should have clean face and hair, be well-dressed, stylish and his clothes should be clean and tidy.

 In relation to physicians’ ethics, he enumerates the characteristics that a physician should have; for example, that the physician should be open-minded, smiling and eloquent. *Rhazes* also adds that the doctor should not be bad tempered in hasty work and should be fearless in treatment.

 Regarding the doctor’s treatment of the patient, *Rhazes* says that the doctor should be a good speaker, of high position, kind and compassionate to the patient and keep the patient’s secret. He should stay with the patient while taking blood and giving medicine. The doctor should not be so harsh that the patient does not like him and should not be too friendly to be considered insignificant but should be in the eyes of the patient, precious and high-minded so that the patient obeys him. *Rhazes* also notes that the doctor should not be greedy for money and possessions.

*Rhazes* believed that anyone could be wiser and superior than Hippocrates at any time but those who follow the whims and desires of their hearts cannot become trained and knowledgeable physicians and everyone is called a knowledgeable physician when his righteousness and correct opinions is more than the slips and inaccuracies of his view and the fewer his medical errors, the greater his wisdom.^31^

*Rhazes* has also taken a brief look at the doctor’s personal life and the way he lives, and says about this:

*“It is better to look at a doctor’s past life and check his efforts in solitude. If he has spent his life flipping through the books of doctors and biologists and he tries to study their work while being alone*,* we should look at his life with a good view. If the passage of his life has not been what we have mentioned and his effort was playing and drinking wine when he was alone, we should look at him with a bad view”*.^31^

## Theoretical Part

 The theoretical part is like an in-depth interview that evaluates the knowledge and attitude of physicians, in the section of cognition of physician knowledge, the basics of medicine are measured and the focus is more on the amount of study and his mastery in medicine. He considers specialized questions about the anatomy and physiology of the organs which include the function and efficiency of the organs, as one of the first questions in this section and the examiner should consider whether the physician has the ability to measure, and if he can understand the books of his predecessors. In the end, he points out that if the therapist does not have this ability, he will no longer need to be tested in examining the patient and identifying the patient but if he has knowledge of the above questions, you can now test his effectiveness in recognizing the disease at the patient’s bedside.^32^

*Rhazes* says it is better to ask questions related to pharmacology in the pharmacy sector but the doctor’s test with knowledge of drugs should be simple because this test is more worthy for a pharmacist than for a doctor unless his knowledge is less than commonly used drugs.

 In the extent to which physicians read and have access to books and articles by experts in medicine and semiotics and cognition of disease, he points out that one should ask the physician “in which books have Hippocrates and others spoken about the background symptoms of cognition of diseases and proper treatment? If the answer is correct, one should ask what was the opposing and agreeing views of the ancient physicians about them? He also knows the conditions of a good doctor being constantly with the book and accompanying the speakers and debaters and assessing this point whether he has understood what he has read or not? And what he has read and understood, has he dealt with the patient? And has he examined the patient? Have other doctors seen his case? Is this disease under his study one of the many diseases or not?^31^

*Mohammad ibn Zakaria al-Rhazes* believed that a physician should also be familiar with other sciences in order to treat various diseases including knowledge of geometry, and astronomy. He considered it necessary for a physician because he would know how to divide times and the state of lands. The physician also needs to know the knowledge of logic otherwise he will be incapable of dividing the type of diseases into his subgroups and he will not be able to understand the correctness and the error of the work in how to plan treatment and follow up.

 Regarding the tests that should be taken from the doctor, from *Rhaze’s* point of view, most questions should be about the fever of severe diseases and the crisis and its days. Also, ask about the difference between different types of fever based on the first period, and how to diagnose combination fever which is considered to be a sign of the doctor’s attention and on the other hand is a sign of his wisdom and knowledge. He also adds that doctors should be tested for recognizing different forms of the disease and separating them from each other. The physician must be able to distinguish between the pain of similar diseases such as pain of colon disease and pneumonia. He should also be able to isolate diarrhea caused by the liver as it occurs from intestinal ulcers and a doctor who is capable of healing severe pain, internal organs and muscles, ligation and contraction of the extremities, cancerous wounds, obsessive-compulsive disorder, malignant wounds, gout, sciatica, epilepsy, paralysis, laxity, partial headache, dizziness, high-fat pus trapped in the chest space, asthma, bleeding from the lungs, a gut that is unable to hold digested food, intestinal ulcers, inflammation of the internal organs and if he improves all of them with medication and an oral program, he is considered as a trained physician.^31^

 Another topic that the examiner should address is the signs of mood and the temperament of the organs that should be asked. The nature of foods and medicines should also be tested and the four periods of illness and treatment in each of these times should be questioned as well as the type of disease because these are information that no one can access medical science except by knowing them correctly.

## Practical Part

 In addition to the above, *Rhazes* also considered the practical aspect of the test and considers this section to include different types of pulse and urology. He believed that achieving this knowledge is through experimentation, experience and measurement (analogy).

 In relation to pulse-ology, several criteria have been considered to measure this section:

- Separating between strong pulse and hard pulse
- Distinguishing between small pulse and weak pulse
- Perception of uniform pulse and different pulse

Regarding the diagnosis of the disease from urine, the physician should be asked about the consistency, color, taste and smell of urine. The question must also be asked about its nature, what it separates from and how and where it gets colored. The physician should be aware of how the urine settles and its types.

 In addition to the above, *Rhazes* is not unaware of the examiner and mentions some characteristics for him: “A person taking the oral test must be eloquent and knowledgeable, and be aware of eloquence but in a clinical examination if such a person is not available, there will be no need and an expert examiner should test the physician about pulse science”.^31^

 The appropriate conditions and necessities in the field of medicine for physicians were not hidden from the view of physicians before and after *Rhazes*, and they have also pointed out that they have these conditions and observe their rules. *Ibn Raban Tabari* (783-858 AD), philosopher, psychologist and physician of the second and third centuries AH in his book *Ferdows al- Hikma* considers intelligence, handsome, decency, sobriety, kindness, patience and generosity necessary for medical students and forbids them from selfishness, jealousy, lies, malice and slander. *Ibn Rabban* believed that it is better for a physician not to rush into treatment and not to treat anyone until he has acquired the skills and training and knowledge of medicines, because medicine is like a terrible poison in unskillful hands.^33^

 In this study, short sections of the recommendations of scientists and physicians after *Rhazes* regarding individual and social features and theoretical and practical parts of medical science are given to clarify the concerns of other physicians in the importance of the subject.

*Ali Ibn Abbas Ahwazi* (930-994 AD) writes in the introduction to *Kamil al- Sina’a al- Tabiyya*: *“The physician should do well and be eloquent. It is incumbent upon the physician to be kind, chaste, friendly*,* a lover of good deeds and popular, and eager to treat the patients, especially the poor and not to expect rewards in return”*.^34^ One of the things that are obligatory for the students of this profession is that he should regularly visit hospitals and sanatoriums and take full care of patients. This care should be done along with the help of very skilled medical professors.^4^

 According to *Avicenna* (980-1080 AD), the first condition of medicine is scientific mastery and knowledge of the patient’s condition and recognition of the disease. He says: *“The physician should have complete information and scientific and practical mastery about the elements of diseases*,* temperaments, simple and complex organs, soul and psyche, natural forces, vital and psychic, actions, health and disease states, environmental factors, food and drink”*.^35^

*Seyed Ismail Jorjani* (1043-1137 AD), the famous physician of the sixth century AH, insists on two important principles of medical ethics, namely to benefit the patient and not to harm him.^36^ *Jorjani* believes that a physician should be present at the patient’s bedside when he fulfills the conditions of religious trust and compassion of the people and keeps his eyes, ears, hands and tongue from all the unpleasant things.^37^

## Conclusion

 In conclusion, evaluating and determining the professional qualifications of physicians is one of the important aspects of medical education and healthcare systems in different countries of the world. This issue is understandable considering the oath that this group takes at the beginning of their career and the undeniable impact of physicians’ qualification on various aspects of the health system, so professional competence is one of the key points emphasized in the “Charter of Commitment to Professional Obligations” published by the Board and the American Internal Medicine Association in 1995. Also, the competence of the medical profession is an important issue in medical research and many studies have been conducted with the aim of providing effective tests to evaluate the competence of the profession of physicians and therapists.^38,39^

 During the 20th century, there has been a return to the moral beliefs of the Hippocratic Oath in a progressively increasing number of medical schools in the Western world as an integral part of professionalization. In 1928, only 13 medical schools in US used the Oath or some version of it,^40^ while the number increased to 51 in 1977 and 69 in 1993.^41^

 Nowadays, in about half of the medical schools in the United Kingdom, in almost all of the medical schools in the United States, and in the majority of Western medical schools students take some form of an oath, usually at graduation.^41,42^ Undoubtedly, the Hippocratic Oath has endured until today because it was the first to put forth the moral obligation of the physician to practice humane patient-oriented medicine.

 The results of the present study show that *Rhazes* is one of the physicians of the Islamic civilization period who in his book *Al- Hawi Fi Al- Tibb* has dealt with all aspects of individual and social ethics and theoretical and practical parts of treatment (Table 1). The necessity of this compels *Rhazes* to compile a comprehensive and structured test with the aim of determining the qualifications of physicians to provide a tool for evaluating the knowledge, attitudes, behaviors and various skills of physicians to the scientific community of their time. This book contains many useful points, from the smallest issues related to the doctor and its relationship with the patient to the personal issues and habits and life of a doctor have been investigated, some of which are more thought-provoking and useful after hundreds of years. Although a number of scientists and physicians before and after *Rhazes* have referred in their books to the subject of testing and passing medical procedures in order to determine the qualifications for medicine, none of them like *Rhazes* has discussed in a coherent and purposeful way about testing physicians. It is necessary for physicians and medical students to put the ethical, behavioral and professional issues mentioned by this great scientist in his valuable book *Al- Hawi Fi Al- Tibb* as the headline of their medical profession by observing all the rules and principles to better identify the high status of medical science and the relationship between patient and physician.

**Table 1:** Evaluation of Determining the Competence of Physicians from *Rhazes* Point of View in the Book *Al- Hawi Fi Al- Tibb*

**No**	**Title**	**Description**
1	Examining personal life	- Has he spent his life studying and reading medical books and testing or done other things? - Does he spend his time in solitude and free time in experimenting and trying in the field of medicine or spend time playing, having fun and drinking wine? - Has he always been the companion of speakers and debaters and has he benefited from them?
2	Physicians treatment skills	- Choosing the right treatment method - Accurate knowledge of diseases and their isolation - Observing the condition of the poor and needy when prescribing medicine - Familiarity with natural sciences, geometry, astronomy, logic and mathematics - He has to be eloquent and theorist - He has to be aware of the patient's internship and nursing
3	Physical features	- He should be agile - Should have clean face and hair - Should be well-dressed, stylish and tidy
4	Moral and behavioral characteristics	- Should be cheerful and smiling - Should be well-thought and well-spoken - Should be smart and tactful - Should be not be bad tempered in hasty work - Should be fearless in treatment - Should be not be greedy for money and property - Should be high position and middle-aged - Should be kind and compassionate to the patient - Should keep the secrets of his patient - Should stay with the patient while taking blood and giving medicine - Should not be so harsh that the patient does not like him - Should not be too friendly to be considered insignificant but should be in the eyes of the patient, precious and high-minded - Should communicate an emotional connection with his patient, as this will help him better identify the disease
5	Theoretical test	- The physician should be asked in which books have Hippocrates and others spoken correctly about the background and symptoms of illness and treatment? - Does he read and use the books of Hippocrates? - Has he studied the books of philosophers and is he able to discuss and comment on their opinions or not? - Is he able to understand the books of the predecessors? - Does he have the ability of measuring? - Should know the opposing and agreeing views of former physicians about diseases - He should be asked about the anatomy and physiology of the organs - He must know the signs of temperament and temperament of the organs - Should know the nature of foods and medicines - Should Know Times of quadruple of the diseases and the treatment methods of each of them
6	Practical test	- Recognition of the disease on the patient's bedside - He should be tested in pulse-ology and should know the types of pulses - Should know and describe the types of urine - He should be asked about the fever of severe illnesses and about the crisis and its days - He should be asked about distinguishing between different types of combination fevers
